# Exploring the Relationship Between Medication Adherence and Diabetes Disparities among Hispanic Patients in a Large Health System

**DOI:** 10.1007/s11606-023-08502-y

**Published:** 2023-11-14

**Authors:** Kimberly Danae Cauley Narain, Ayan Patel, Samuel Skootsky, Carol M. Mangione

**Affiliations:** 1 Division of General Internal Medicine and Health Services Research, Department of Medicine, David Geffen School of Medicine, University of California, Los Angeles, CA USA; 2 Center for Health Advancement, Fielding School of Public Health, University of California Los Angeles, Los Angeles, CA USA; 3 Center for Data-Driven Insights & Innovation, University of California Health, Oakland, CA USA; 4 Population Health, University of California Health, Oakland, CA USA; 5 Fielding School of Public Health, University of California, Los Angeles, CA USA

**Keywords:** diabetes, disparities, medication adherence, Hispanics

## Abstract

**Background:**

Sub-optimal HbA1c control is a driver of disparities in diabetes outcomes among Hispanic patients. Differences in medication adherence may underlie racial/ethnic differences in HbA1c level.

**Objective:**

To examine the relationship between medication adherence and disparities in HbA1c level among Hispanic patients, relative to other racial/ethnic groups, obtaining care in the University of California Health System (UC Health).

**Design:**

This study used clinical, administrative, and prescription dispensing data (January–December 2021) derived from the electronic health records of 5 Academic Medical Centers in UC Health, and linear regression models (LRMs) to conduct a cross-sectional analysis of the association between medication adherence, race/ethnicity, and HbA1c level. Adjusted LRMs were run with and without the measure of medication adherence to assess this relationship.

**Participants:**

Patients with a UC Health primary care physician (PCP), with ≥ 1 PCP visit within the last 3 years, ages 18–75, reporting Asian, Hispanic, or White race/ethnicity, and who had ≥ 2 encounters with an ICD diagnosis of diabetes or had a prescription for a diabetes medication within the last 2 years, as of 12/31/21 (*N* = 27, 542; Asian = 6253, Hispanic = 7216, White = 14,073).

**Main Measures:**

Our measure of medication adherence was the proportion of days covered (PDC) for diabetes medications in 2021. Our outcome was the most recent HbA1c value.

**Key Results:**

In the LRM excluding the PDC, Hispanic ethnicity was positively associated with HbA1c level (β = 0.31, *p* =  < 0.001). In the LRM model including PDC, PDC was negatively associated with HbA1c level (β =  − 0.18, *p* =  < 0.001). However, the positive relationship between Hispanic ethnicity and HbA1c level did not change (β = 0.31, *p* =  < 0.001).

**Conclusions:**

The findings of this study suggest that the relationship between Hispanic ethnicity, HbA1c level, and factors outside of medication adherence should be explored among primary care patients receiving care in Academic Medical Centers.

**Supplementary Information:**

The online version contains supplementary material available at 10.1007/s11606-023-08502-y.

## INTRODUCTION

Hispanic Americans bare a disproportionate share of the diabetes burden. The prevalence of type 2 diabetes (T2DM) among Hispanic individuals is significantly higher than the prevalence among non-Hispanic White (hereafter, White) individuals (11.8% vs. 7.4%).^1^ Relative to White individuals, Hispanic individuals with T2DM are more likely to develop end-stage renal disease and retinopathy and require lower extremity amputations.^2^ Furthermore, the prevalence of T2DM is increasing at a faster rate among Hispanic individuals, compared to that of White individuals.^2^

Higher rates of uninsurance among Hispanic individuals, relative to White individuals, is an important driver of differential T2DM outcomes (21% vs. 7%).^3^ However, even after taking health insurance coverage into account, disparities in HbA1c control and other cardiovascular disease risk factors among Hispanic patients with T2DM persist. Heisler et al. (2007) analyzed data from the 2003 diabetes supplement to the Health and Retirement Study and found a 0.74% difference in HbA1c across Hispanic and non-Hispanic patients, after adjusting models for health insurance coverage.^4^ Among the factors contributing to persistent disparities among Hispanic patients with T2DM is lower levels of treatment intensification, relative to White patients. Using NHANES data (2003–2012), Perez et al. (2015) found that Mexican individuals with uncontrolled T2DM, relative to White individuals, were less likely to have regimens intensified to non-insulin triple therapies (7.3% vs 11.3%) or insulin-based therapies (23.7% vs 30.5%) and were more likely to not be on any medications (17.2% vs 10.4%).^5^ Another potential contributor to worse T2DM outcomes among Hispanic individuals, relative to White individuals, is reduced adherence to diabetes medications. Yang et al. (2009) found a 37% higher risk of reporting non-adherence to diabetes medications among Hispanic patients with T2DM on Medicare Part D, relative to White patients.^6^ The factors underlying less treatment adherence among Hispanic individuals, relative to White individuals, are diverse and multi-level and include negative health beliefs about diabetes medications, lower individual, and neighborhood-level socioeconomic status on average and a higher proportion of individuals with limited English proficiency.^2, 7^

There are a number of gaps in our understanding of the relationship between medication adherence and racial/ethnic differences in HbA1c level among patients with diabetes. To our knowledge, no prior studies have explored the relationship between medication adherence and racial/ethnic differences in HbA1c level among patients with diabetes within a large academic health system context. Exploring this relationship using electronic health record data builds on prior knowledge by allowing for considerations of different factors that may have confounded previous survey-based results such as access to specialty care, diabetes severity, and co-morbidity. Additionally, prior analyses have relied on self-reported measures of medication adherence, which may be less valid than claims-based measures of adherence. Lastly, the most recent studies on this topic have relied on data that is more than a decade old. As such, it is worth revisiting this topic using data from a time period that is more representative of the current landscape of treatment options for diabetes. We endeavored to address some of these gaps in the literature by exploring the relationship between medication adherence and disparities in HbA1c level among Hispanic patients with diabetes, relative to other racial/ethnic groups, obtaining care in a large California-based academic health system. The study hypotheses were that medication adherence would be negatively associated with HbA1c level, controlling for potential confounders, and medication adherence would account for some of the disparity in HbA1c level observed among Hispanic patients.

### Setting

The University of California Health System (UC Health) is the largest academic health system and the 12th largest health system in the USA, as estimated by the number of affiliated physicians.^8^ UC Health consists of six academic medical centers (AMCs) located in geographically distributed urban areas in California with more than 8.1 million outpatient visits in 2020, providing care for roughly 1.8 million unique patients.^9^ California ranks first in linguistic diversity and second in racial/ethnic diversity among states.^10^ There is also substantial variability in the demographic profile of the patient populations served across each of UC Health’s constituent AMCs. In 2021, the Population Health Program (an entity with representation from all constituent AMCs, focused on bolstering value-based care delivery, improving patient outcomes, lowering cost, and improving health equity within the UC Health patient population) identified worse HbA1c control among Hispanic primary care patients, relative to other racial/ethnic groups (American Indian/Alaskan Natives, Asian, Black, White). Consequently, the Population Health Program team was interested in exploring factors that might be underlying this racial/ethnic difference.

### Study Design

The data set comprises health data (27,542 patient records) across 5 of the 6 AMCs (January–December of 2021), in the University of California (UC) Health system. Institutional Review Board approval requirements are stipulated by the constituent academic medical centers. Because the focus of this work was quality improvement, IRB approval was not required by UCLA. All methods were carried out in accordance with relevant university guidelines and regulations.

### Data Source

The dataset consist of selected clinical and administrative data from the electronic health records of 5 of the 6 AMCs (January–December of 2021), including information on medical encounter types, demographics, ICD codes, CPT codes, vital signs, laboratory test results, health insurance coverage, prescription medication orders and prescription dispensing data supplemented with data derived from the Healthy Places Index (HPI), and a California-specific index constructed using census-level data covering economic, education, healthcare access, housing, neighborhood, clean environment, transportation, and social environmental domains.^11, 12^ The 6th AMC was newly added to Population Health Program and unable to transmit complete patient data in time to be included in the analyses.

### Study Sample

The study sample consisted of patients with a UC Health primary care physician (PCP), with ≥ 1 PCP visit within the last 3 years, ages 18–75, self-reporting Asian, Hispanic, or White race/ethnicity through the electronic health record, and who had ≥ 2 encounters with an ICD diagnosis of type 2 diabetes or had a prescription for a diabetes medication within the last 2 years, as of 12/31/21. Individuals on metformin without additional evidence of a diabetes diagnosis (e.g., ICD code or additional diabetes medication) were excluded due to other clinical uses for metformin outside of diabetes such as pre-diabetes. The study population was limited to White patients (the largest racial/ethnic subgroup in the UC Health), Hispanic patients (statistically significant lower HbA1c control relative to White patients), and Asian patients (statistically significant higher HbA1c control relative to Hispanic and White patients). Other racial/ethnic groups without statistically significant differences in HbA1c control from those of White patients were excluded in effort to limit the number of variables included in the model and to reduce some of variability across the study population that was not germane to the research question.

### Measures

The measure of medication adherence was the proportion of days covered (PDC) (the proportion of days in the treatment period “covered” by prescription claims for the same medication or another in its therapeutic category).^13^ In accordance with the HEDIS recommendation for the diabetes PDC, the classes of diabetes medications included in the PDC measure were biguanides, sulfonylureas, thiazolidinediones, or DPP-IV inhibitors, GLP-1 receptor agonists, meglitinides, and sodium glucose co-transporter 2 inhibitors.^13^ The outcome was the most recent HbA1c value which was treated as a continuous variable. Covariates were considered for inclusion in the model based on review of conceptual models of diabetes medication adherence in the literature.^14^ Covariates with statically significant differences across race/ethnicity in bivariate analyses were retained in the models. The variables included in the mode were race/ethnicity (Asian, Hispanic and White (reference group)), age group (65 +  = reference group), gender, marital status, preferred language (English vs. Non-English), insurance type (private = reference, Medicare, Medicaid, Medicare + Medicaid), smoking status, the Adapted Diabetes Complications Severity Index (aDCSI)^15^, a count of co-morbidities often associated with diabetes (sleep apnea, hypertension and obesity), a count of endocrinologist visits in the last year, an indicator for depression, an indicator for neighborhood disadvantage (living in the highest HPI quartile), and AMC fixed-effects (reflects stable structural differences across AMCs). All clinical conditions were identified using ICD codes.

### Statistical Analysis 

“Test of proportions” and *t*-test were used to conduct bivariate analyses of covariates and outcomes across Asian and Hispanic patients and across Hispanic and White patients for dichotomous and continuous variables, respectively. For the adjusted analyses, first, an ordinary least squares (OLS) regression model of HbA1c level including all covariates with the exception of PDC was run. Next, the same OLS model was run including the PDC measure. The point estimates were beta coefficients and statistical significance was determined at the *p*-value < 0.05 level. Missing data was dealt with via complete case analysis. Two sensitivity analyses were run, one including the number of primary care visits in the last year in the models with and without PDC control and another one limiting the study population to the subpopulation with a baseline HbA1c ≥ 7%.

## RESULTS

The study sample consisted of 27,542 patients (Asian = 6253, Hispanic = 7216, White = 14,073). The mean PDCs and average baseline HbA1c values were 90.03%, 86.40%, 88.83%, and 7.08%, 7.65%, and 7.21% for Asian, Hispanic, and White patients, respectively. Table 1 highlights the results of the bivariate analyses. In bivariate analyses comparing Asian to Hispanic patients, Hispanic patients had a lower PDC, were younger, were less likely to have English as a preferred language, were less likely to be married, were more likely to have a depression diagnosis, were more likely to smoke, had a higher mean aDSI, and had a mean number of endocrinology visits, were more likely to have Medicaid/Medicaid + Medicare insurance, were less likely to have exclusive Medicare, were and more likely to live in a highly disadvantaged neighborhood. In comparing Hispanic to White patients, Hispanic patients had a lower PDC, had skewed younger, were more likely to be female, were less likely to have English as a preferred language, were less likely to be married, were less likely to smoke, had a higher mean aDSI, had a lower mean number of endocrinology visits and primary care visits, were more likely to have Medicaid/Medicaid + Medicare insurance, were less likely to have exclusive Medicare, and were more likely to live in a highly disadvantaged neighborhood.

**Table 1:** Bivariate Analyses Across Race/Ethnicity

Covariates	Asian^1^	*p*-values	Hispanic^2^	*p*-values	White^3^
Baseline HbA1c (SD)	7.08 (1.37)	< 0.001	7.65 (1.84)	< 0.001	7.21 (1.58)
Proportion of days covered (SD)^4^	90.03 (16.34)	< 0.001	86.40 (19.50)	< 0.001	88.83 (17.40)
18–35	4.04%	< 0.001	8.67%	< 0.001	6.81%
36–50	17.71%	< 0.001	20.96%	< 0.001	11.44%
51–64	34.34%	< 0.001	38.04%	< 0.001	33.59%
65 +	43.90%	< 0.001	32.32%	< 0.001	48.17%
Female	51.48%	0.53	51.97%	< 0.001	43.31%
English primary language	86.67%	< 0.001	72.34%	< 0.001	98.53%
Married	70.88%	< 0.001	56.58%	< 0.001	59.03%
Depression	6.17%	< 0.001	12.70%	0.81	12.60%
Smoking	3.58%	< 0.001	5.03%	< 0.001	6.31%
Diabetes severity index (SD)^5^	0.44 (0.71)	< 0.001	0.53 (0.83)	< 0.001	0.48 (0.70)
Comorbidity count (SD)^6^	0.77 (0.79)	< 0.001	1.05 (0.85)	0.56	1.04 (0.90)
Number of endocrinology visits (SD)	0.83 (1.57)	< 0.001	0.96 (1.67)	0.003	1.02 (1.71)
Medicare	17.49%	< 0.001	11.67%	< 0.001	22.79%
Medicaid	7.03%	< 0.001	13.49%	< 0.001	4.87%
Medicare + Medicaid	4.27%	< 0.001	7.20%	< 0.001	3.53%
Neighborhood disadvantage	5.67%	< 0.001	21.42%	< 0.001	5.33%
Number of PCP visits (SD)	2.25 (2.20)	0.78	2.24 (2.72)	0.001	2.36 (2.64)

The data source is electronic health records from 5 academic medical centers (January–December of 2021). Our study sample consist of patients with a UC Health primary care physician (PCP), with ≥ 1 PCP visit within the last 3 years, ages 18–75, reporting Asian, Hispanic, or White race/ethnicity, and who had ≥ 2 encounters with an ICD diagnosis of diabetes or had a prescription for a diabetes medications within the last 2 years as of 12/31/21. Bivariate analyses are conducted using “test of proportions” and “*t*-test” for dichotomous and continuous variables, respectively. Values and standard deviations are reported for continuous outcomes. Percentages are reported for dichotomous variables

^1^*N* = 6253

^2^*N* = 7216

^3^*N* = 14,073

^4^Proportion of days covered includes all diabetes medications used from January through December of 2021

^5^The Adapted Diabetes Severity Index is the sum of indicator variables for atherosclerotic cardiovascular disease, chronic kidney disease, neuropathy, and retinopathy based on ICD codes. The co-morbidity count was the sum of indicator variables for sleep apnea, hypertension, and obesity based on ICD codes

In the adjusted analyses excluding the PDC (Table 2), the point estimate for Hispanic ethnicity was (β = 0.31, *p* =  < 0.001). This point estimate indicates that reporting Hispanic ethnicity is associated with 0.31 increase in most recent HbA1c, relative to reporting White race, controlling for other factors. After inclusion of the PDC which had a negative and significant association with HbA1c level (β =  − 0.18, *p* =  < 0.001), the point estimate for Hispanic ethnicity (β = 0.31, *p* =  < 0.001) did not change (Table 3). The point estimates for other variables included in the model with the exception of Asian ethnicity did not change appreciably in response to including PDC in the model. The point estimate for Asian ethnicity went from being negative and significant (β =  − 0.07, *p* = 0.02) to no longer statistically significant (β =  − 0.05, *p* = 0.11) in the model controlling for PDC. Female gender, English language preference, being married, a diagnosis of depression, co-morbidity count, exclusive Medicare coverage, and Medicare + Medicaid coverage were associated with a lower HbA1c level. Conversely, age younger than 65, current smoking, a higher aDSI, number of endocrinology visits, neighborhood disadvantage, and AMC 1–3 were associated with a higher HbA1c level. In the models controlling for the number of primary care physician (PCP) visits (Tables 4 and 5), more PCP visits were associated with a lower HbA1c level; however, the point estimate for Hispanic ethnicity remained positive and significant in models with and without PDC control. In the models limited to the subpopulation with a baseline HbA1c ≥ 7% (Supplementary Tables 1–2), the positive association between Hispanic ethnicity and HbA1c level persisted in models with and without PDC control.

**Table 2:** Relationship of Hispanic Ethnicity with HbA1c Level Adjusting for Other Factors

	β	std err	z	*p*-value
Asian	− 0.07	0.03	− 2.25	0.02
Hispanic	0.31	0.03	10.39	< 0.001
18–35	0.31	0.05	5.64	< 0.001
36–50	0.29	0.04	7.56	< 0.001
51–64	0.21	0.03	7.36	< 0.001
Female	− 0.11	0.02	− 4.69	< 0.001
English	− 0.10	0.04	− 2.53	0.01
Married	− 0.12	0.03	− 5.02	< 0.001
Depression	− 0.10	0.04	− 2.81	0.005
Smoker	0.29	0.05	5.70	< 0.001
Diabetes severity index^1^	0.07	0.02	4.45	< 0.001
Comorbidity count^2^	− 0.04	0.01	− 3.06	0.002
Number of endocrinology visits	0.06	0.007	8.60	< 0.001
Medicare	− 0.13	0.03	− 3.71	< 0.001
Medicaid	− 0.06	0.04	− 1.36	0.18
Medicare + Medicaid	− 0.15	0.06	− 2.76	0.006
Neighborhood disadvantage	0.21	0.04	5.40	< 0.001
AMC 1	0.13	0.04	3.21	0.001
AMC 2	0.27	0.03	8.69	< 0.001
AMC 3	0.13	0.04	3.45	0.001
AMC 4	− 0.04	0.04	− 1.04	0.30

The data source is electronic health records from 5 academic medical centers (January–December of 2021). Our study sample consist of patients with a UC Health primary care physician (PCP), with ≥ 1 PCP visit within the last 3 years, ages 18–75 (65 + is the reference group), reporting Asian, Hispanic, or White race/ethnicity (reference group), and who had ≥ 2 encounters with an ICD diagnosis of diabetes or had a prescription for a diabetes medication within the last 2 years as of 12/31/21. *N* = 27,542

*AMC* Academic Medical Center

^1^The Adapted Diabetes Severity Index is the sum of indicator variables for atherosclerotic cardiovascular disease, chronic kidney disease, neuropathy, and retinopathy based on ICD codes. The co-morbidity count was the sum of indicator variables for sleep apnea, hypertension, and obesity based on ICD codes. Private insurance is the reference group for insurance coverage. The statistical model is a linear regression model

**Table 3:** Relationship of Hispanic Ethnicity with HbA1c Level Adjusting for Medication Adherence and Other Factors

	β	std err	*z*	*p*-value
Asian	− 0.05	0.03	− 1.60	0.11
Hispanic	0.31	0.03	10.41	< 0.001
18–35	0.25	0.06	4.64	< 0.001
36–50	0.26	0.04	6.80	< 0.001
51–64	0.20	0.03	6.98	< 0.001
Female	− 0.11	0.02	− 4.85	< 0.001
English	− 0.10	0.04	− 2.50	0.01
Married	− 0.12	0.03	− 4.90	< 0.001
Depression	− 0.10	0.04	− 2.97	0.003
Smoker	0.29	0.05	5.73	< 0.001
Diabetes severity index^1^	0.05	0.02	3.37	0.001
Comorbidity count^2^	− 0.03	0.01	− 2.46	0.01
Number of endocrinology visits	0.05	0.007	7.67	< 0.001
Medicare	− 0.13	0.03	− 3.73	< 0.001
Medicaid	− 0.06	0.04	− 1.44	0.15
Medicare + Medicaid	− 0.15	0.06	− 2.78	0.005
Neighborhood disadvantage	0.21	0.04	5.35	< 0.001
AMC 1	0.13	0.04	3.16	0.002
AMC 2	0.26	0.03	8.61	< 0.001
AMC 3	0.12	0.04	3.34	0.001
AMC 4	− 0.04	0.04	− 1.07	0.28
Proportion of days covered	− 0.18	0.02	− 7.37	< 0.001

The data source is electronic health records from 5 academic medical centers (January–December of 2021). Our study sample consist of patients with a UC Health primary care physician (PCP), with ≥ 1 PCP visit within the last 3 years, ages 18–75 (65 + is the reference group), reporting Asian, Hispanic, or White race/ethnicity (reference group), and who had ≥ 2 encounters with an ICD diagnosis of diabetes or had a prescription for a diabetes medication within the last 2 years as of 12/31/21. *N* = 27,542

*AMC* Academic Medical Center

^1^The Adapted Diabetes Severity Index is the sum of indicator variables for atherosclerotic cardiovascular disease, chronic kidney disease, neuropathy, and retinopathy based on ICD codes

^2^The co-morbidity count was the sum of indicator variables for sleep apnea, hypertension, and obesity based on ICD codes. Private insurance is the reference group for insurance coverage. The statistical model is a linear regression model

**Table 4:** Relationship of Hispanic Ethnicity with HbA1c Level Adjusting for Other Factors with PCP Visit

	β	std err	*z*	*p*-value
Asian	− 0.06	0.03	− 2.11	0.035
Hispanic	0.32	0.03	10.42	< 0.001
18–35	0.29	0.05	5.40	< 0.001
36–50	0.28	0.04	7.37	< 0.001
51–64	0.21	0.03	7.14	< 0.001
Female	− 0.10	0.02	− 4.52	< 0.001
English	− 0.10	0.04	− 2.46	0.014
Married	− 0.12	0.03	− 5.05	< 0.001
Depression	− 0.08	0.04	− 2.29	0.022
Smoker	0.29	0.05	5.73	< 0.001
Diabetes severity index^1^	0.08	0.02	4.75	< 0.001
Comorbidity count^2^	− 0.03	0.01	− 2.33	0.02
Number of endocrinology visits	0.06	0.01	8.36	< 0.001
Medicare	− 0.12	0.03	− 3.64	< 0.001
Medicaid	− 0.06	0.04	− 1.27	0.20
Medicare + Medicaid	− 0.14	0.06	− 2.61	0.01
Neighborhood disadvantage	0.21	0.04	5.39	< 0.001
AMC 1	0.12	0.04	2.97	0.003
AMC 2	0.27	0.03	8.92	< 0.001
AMC 3	0.11	0.04	3.04	0.002
AMC 4	− 0.04	0.04	− 1.14	0.26
Number of PCP visits	− 0.01	0.01	− 3.20	0.001

The data source is electronic health records from 5 academic medical centers (January–December of 2021). Our study sample consist of patients with a UC Health primary care physician (PCP), with ≥ 1 PCP visit within the last 3 years, ages 18–75 (65 + is the reference group), reporting Asian, Hispanic, or White race/ethnicity (reference group), and who had ≥ 2 encounters with an ICD diagnosis of diabetes or had a prescription for a diabetes medication within the last 2 years as of 12/31/21. *N* = 27,542

*AMC* Academic Medical Center

^1^The Adapted Diabetes Severity Index is the sum of indicator variables for atherosclerotic cardiovascular disease, chronic kidney disease, neuropathy, and retinopathy based on ICD codes

^2^The co-morbidity count was the sum of indicator variables for sleep apnea, hypertension, and obesity based on ICD codes. Private insurance is the reference group for insurance coverage. The statistical model is a linear regression model

**Table 5:** Relationship of Hispanic Ethnicity with HbA1c Level Adjusting for Medication Adherence and Other Factors with PCP Visit

	β	std err	*z*	*p*-value
Asian	− 0.04	0.03	− 1.47	0.14
Hispanic	0.32	0.03	10.44	< 0.001
18–35	0.24	0.06	4.42	< 0.001
36–50	0.25	0.04	6.63	< 0.001
51–64	0.20	0.03	6.78	< 0.001
Female	− 0.11	0.02	− 4.69	< 0.001
English	− 0.10	0.04	− 2.43	0.02
Married	− 0.12	0.03	− 4.93	< 0.001
Depression	− 0.09	0.04	− 2.48	0.01
Smoker	0.29	0.05	5.76	< 0.001
Diabetes severity index2^1^	0.06	0.02	3.66	< 0.001
Comorbidity count^2^	− 0.02	0.01	− 1.79	0.07
Number of endocrinology visits	0.05	0.007	7.46	< 0.001
Medicare	− 0.12	0.03	− 3.66	< 0.001
Medicaid	− 0.06	0.04	− 1.36	0.17
Medicare + Medicaid	− 0.15	0.06	− 2.64	0.01
Neighborhood disadvantage	0.21	0.04	5.34	< 0.001
AMC 1	0.12	0.04	2.93	0.003
AMC 2	0.27	0.03	8.82	< 0.001
AMC 3	0.11	0.04	2.95	0.003
AMC 4	− 0.04	0.04	− 1.16	0.24
Proportion of days covered	− 0.17	0.02	− 7.30	< 0.001
Number of PCP visits	− 0.01	0.005	− 3.01	0.003

The data source is electronic health records from 5 academic medical centers (January–December of 2021). Our study sample consist of patients with a UC Health primary care physician (PCP), with ≥ 1 PCP visit within the last 3 years, ages 18–75 (65 + is the reference group), reporting Asian, Hispanic, or White race/ethnicity (reference group), and who had ≥ 2 encounters with an ICD diagnosis of diabetes or had a prescription for a diabetes medication within the last 2 years as of 12/31/21. *N* = 27,542

*AMC* Academic Medical Center

^1^The Adapted Diabetes Severity Index is the sum of indicator variables for atherosclerotic cardiovascular disease, chronic kidney disease, neuropathy, and retinopathy based on ICD codes. The co-morbidity count was the sum of indicator variables for sleep apnea, hypertension, and obesity based on ICD codes. Private insurance is the reference group for insurance coverage. The statistical model is a linear regression model

## DISCUSSION

Using EHR data from a large academic health system in California and a cross-sectional study design, this study found that Hispanic ethnicity was associated with lower adherence to diabetes medications, relative to other racial/ethnic groups and that PDC was negatively associated with HbA1c level. However, controlling for PDC in adjusted models did not reduce the positive association between Hispanic ethnicity and HbA1c level, suggesting that medication adherence is not the main driver of racial/ethnic differences in HbA1c level, among this study population. Controlling for access to primary care or restricting the study population to individuals with a baseline ≥ 7% did not change the relationship between Hispanic ethnicity, PDC, and HbA1c level. Consequently, there is only partial support for the study hypotheses. The finding of lower adherence to diabetes medications among Hispanic patients, relative to other racial/ethnic subgroups of patients in this study, is consistent with the work of Yang et al. (2009).^6^ This study makes several contributions to the literature such as controlling for a wider range of potential confounders, relative to survey-based studies (access to primary and specialty care, diabetes severity, co-morbidity, and diabetes severity), using a more valid measure of medication adherence, relative to self-report, and studying this topic using relatively recent data. However, there are important study limitations that must be considered such as a lack of controls for individual-level socioeconomic status, inability to account for differences in health literacy, lack of measures of other health behaviors (e.g., dietary quality and physical activity), and the absence of measures of care quality. The measure of medication adherence (PDC) reflects the possession of diabetes medications rather than taking them at all or taking them properly. Lastly, we lack of data on the distribution of 30- vs. 90-day medication supply across race/ethnicity which could potentially confound these results. Future studies should explore the role of lifestyle modification, individual-level socioeconomic status, and healthcare quality in HbA1c disparities among Hispanic patients within an academic medical center context.

## CONCLUSIONS

Using EHR data from a large California-based academic health system and a cross-sectional study design, this study explored the relationship between adherence to diabetes medications and racial/ethnic disparities in HbA1c level. This study found that medication adherence was negatively associated with HbA1c level. However, medications adherence did not explain the positive association between Hispanic ethnicity and HbA1c level. The role of factors outside of medication adherence such as diet, exercise, socioeconomic status, and care quality in higher HbA1c levels among Hispanic patients should be explored.

### Supplementary Information

Below is the link to the electronic supplementary material.

Supplementary file1 (DOCX 17 KB)

## Data Availability

This study uses clinical operations data in the context of a quality improvement project. As such, this data is not publicly available due to HIPAA compliance requirements and UCLA IRB regulations.
