# Supplementary material for: Exploring the Relationship Between Medication Adherence and Diabetes Disparities among Hispanic Patients in a Large Health System
Source: J Gen Intern Med. 2023 Nov 14;39(7):1149–55. doi: 10.1007/s11606-023-08502-y (PMC11116283; doi:10.1007/s11606-023-08502-y)
Supplement: Supplementary file 1 — Supplementary file1 (DOCX 17 KB) [file 11606_2023_8502_MOESM1_ESM.docx]

| **Supplemental Table 1. Relationship of Hispanic Ethnicity With HbA1c Level Adjusting for Other Factors Among Patients with a Baseline HbA1c ≥ 7** | | | | |
| --- | --- | --- | --- | --- |
|  | **β** | **std err** | **z** | ***p*-value** |
| **Asian** | -0.19 | 0.05 | -3.51 | <0.001 |
| **Hispanic** | 0.32 | 0.05 | 6.38 | <0.001 |
| **18-35** | 0.51 | 0.09 | 5.59 | <0.001 |
| **36-50** | 0.48 | 0.07 | 7.41 | <0.001 |
| **51-64** | 0.20 | 0.05 | 4.07 | <0.001 |
| **Female** | -0.10 | 0.04 | -2.55 | 0.01 |
| **English** | 0.04 | 0.07 | 0.55 | 0.58 |
| **Married** | -0.15 | 0.04 | -3.63 | <0.001 |
| **Depression** | 0.03 | 0.06 | 0.42 | 0.67 |
| **Smoker** | 0.23 | 0.08 | 2.81 | 0.005 |
| **Diabetes Severity Index2**^1^ | 0.06 | 0.03 | 2.25 | 0.03 |
| **Comorbidity count^2^** | -0.09 | 0.02 | -4.00 | <0.001 |
| **Number of Endocrinology Visits** | -0.01 | 0.01 | -0.59 | 0.56 |
| **Medicare** | -0.17 | 0.06 | -2.83 | 0.01 |
| **Medicaid** | 0.09 | 0.07 | 1.16 | 0.25 |
| **Medicare+Medicaid** | -0.01 | 0.09 | -0.12 | 0.91 |
| **Neighborhood Disadvantage** | 0.15 | 0.06 | 2.35 | 0.02 |
| **AMC 1** | 0.05 | 0.07 | 0.71 | 0.48 |
| **AMC 2** | -0.06 | 0.05 | -1.10 | 0.27 |
| **AMC 3** | 0.03 | 0.06 | 0.53 | 0.59 |
| **AMC 4** | -0.24 | 0.07 | -3.55 | <0.001 |
| The data source is electronic health records from 5 academic medical centers (January-December of 2021). Our study sample consist of patients with a UC Health primary care physician (PCP), with ≥ 1 PCP visit within the last 3 years, ages 18-75 (65+ is the reference group), reporting Asian, Hispanic, or White race/ethnicity(reference group) , and who had ≥ 2 encounters with an ICD diagnosis of diabetes or had a prescription for a diabetes medication within the last 2 years as of 12/31/21” N=16,785 1. The Adapted Diabetes Severity Index is the sum of indicator variables for atherosclerotic cardiovascular disease, Chronic Kidney Disease, Neuropathy and Retinopathy based on ICD codes. The co-morbidity count was the sum of indicator variables for sleep apnea, hypertension and obesity based on ICD codes. Private insurance is the reference group for insurance coverage. The statistical model is a linear regression model. AMC=Academic Medical Center | | | | |
|  | | | | |

| **Supplemental Table 2. Relationship of Hispanic Ethnicity With HbA1c Level Adjusting for Medication Adherence & Other Factors Among Patients with a Baseline HbA1c ≥ 7** | | | | |
| --- | --- | --- | --- | --- |
|  | **β** | **std err** | **z** | ***p*-value** |
| **Asian** | -0.13 | 0.05 | -2.45 | 0.01 |
| **Hispanic** | 0.33 | 0.05 | 6.43 | <0.001 |
| **18-35** | 0.37 | 0.09 | 4.09 | <0.001 |
| **36-50** | 0.42 | 0.07 | 6.42 | <0.001 |
| **51-64** | 0.17 | 0.05 | 3.42 | 0.001 |
| **Female** | -0.11 | 0.04 | -2.73 | 0.0060 |
| **English** | 0.04 | 0.07 | 0.65 | 0.52 |
| **Married** | -0.15 | 0.04 | -3.49 | <0.001 |
| **Depression** | 0.01 | 0.06 | 0.19 | 0.85 |
| **Smoker** | 0.23 | 0.08 | 2.81 | 0.005 |
| **Diabetes Severity Index2**^1^ | 0.02 | 0.03 | 0.74 | 0.46 |
| **Comorbidity count^2^** | -0.08 | 0.02 | -3.43 | 0.001 |
| **Number of Endocrinology Visits** | -0.02 | 0.01 | -2.09 | 0.04 |
| **Medicare** | -0.17 | 0.06 | -2.86 | 0.004 |
| **Medicaid** | 0.07 | 0.07 | 0.96 | 0.34 |
| **Medicare+Medicaid** | -0.02 | 0.09 | -0.22 | 0.83 |
| **Neighborhood Disadvantage** | 0.14 | 0.06 | 2.27 | 0.02 |
| **AMC 1** | 0.05 | 0.07 | 0.67 | 0.50 |
| **AMC 2** | -0.07 | 0.05 | -1.28 | 0.20 |
| **AMC 3** | 0.01 | 0.06 | 0.19 | 0.85 |
| **AMC 4** | -0.26 | 0.07 | -3.81 | <0.001 |
| **Proportion of Days Covered** | -0.40 | 0.04 | -9.58 | <0.001 |
| The data source is electronic health records from 5 academic medical centers (January-December of 2021). Our study sample consist of patients with a UC Health primary care physician (PCP), with ≥ 1 PCP visit within the last 3 years, ages 18-75 (65+ is the reference group), reporting Asian, Hispanic, or White race/ethnicity(reference group) , and who had ≥ 2 encounters with an ICD diagnosis of diabetes or had a prescription for a diabetes medication within the last 2 years as of 12/31/21”. N=16,785 1. The Adapted Diabetes Severity Index is the sum of indicator variables for atherosclerotic cardiovascular disease, Chronic Kidney Disease, Neuropathy and Retinopathy based on ICD codes. 2.The co-morbidity count was the sum of indicator variables for sleep apnea, hypertension and obesity based on ICD codes. Private insurance is the reference group for insurance coverage. The statistical model is a linear regression model. AMC=Academic Medical Center | | | | |
